# Psychological burden of the COVID-19 pandemic and its associated factors among frontline doctors of Bangladesh: a cross-sectional study

**DOI:** 10.12688/f1000research.27189.3

**Published:** 2021-01-11

**Authors:** Lingkan Barua, Muhammed Shahriar Zaman, Fardina Rahman Omi, Mithila Faruque

**Affiliations:** 1 Department of Noncommunicable Diseases, Bangladesh University of Health Sciences (BUHS), Dhaka, Mirpur, 1216, Bangladesh; 2 School of Rehabilitation Therapy, Queen’s University, Kingston, ON, Canada

**Keywords:** COVID-19, mental health, doctors, risk factors, Bangladesh

## Abstract

**Background: **Frontline doctors are the most vulnerable and high-risk population to get the novel coronavirus disease 2019 (COVID-19) infection. Hence, we aimed to evaluate the anxiety, depression, sleep disturbance and fear of COVID-19 among frontline doctors of Bangladesh during the pandemic, and the associated factors for these psychological symptoms.

**Methods: **In total, 370 frontline doctors who were involved in the treatment of suspected or confirmed COVID-19 patients during the pandemic took part in an online cross-sectional study. Recruitment was completed using convenience sampling and the data were collected after the start of community transmission of COVID-19 in the country. Anxiety and depression, sleep disturbance, and fear of COVID-19 were assessed by the Patient Health Questionnaire-4, two-item version of the Sleep Condition Indicator, and the Fear of Coronavirus-19 scale, respectively. Socio-demographic information, health service-related information, co-morbidity, and smoking history were collected for evaluating risk factors. The proportion of psychological symptoms were presented using descriptive statistics and the associated factors were identified using multinomial logistic regression analysis.

**Results: **Of the doctors, 36.5% had anxiety, 38.4% had depression, 18.6% had insomnia, and 31.9% had fear of COVID-19. In multinomial logistic regression, inadequate resources in the workplace were found as the single most significant predictor for all psychological outcomes: anxiety and/or depression (severe, OR 3.0, p=0.01; moderate, OR 5.3, p=0.000; mild, OR 2.3, p=0.003), sleep disturbance (moderate, OR 1.9, p=0.02), and fear of COVID-19 (severe, OR 1.9, p=0.03; moderate, OR 1.8, p=0.03).

**Conclusions: **The study demonstrated a high burden of psychological symptoms among frontline doctors of Bangladesh during the COVID-19 pandemic situation. Inadequate resources are contributing to the poor mental health of Bangladeshi doctors. The supply of sufficient resources in workplaces and mental health counseling may help to mitigate the burden of the psychological symptoms identified among the respondents..

## Introduction

Novel coronavirus disease 2019 (COVID-19), caused by severe acute respiratory syndrome coronavirus 2 (SARS-CoV-2), was first recognized in December 2019 in Wuhan City in central China ^1, 2^. The World Health Organization declared the COVID-19 outbreak as a global pandemic on March 11, 2020 ^3^. Bangladesh confirmed its first COVID-19 outbreak on March 08, 2020, when the Institute of Epidemiology, Disease Control and Research (IEDCR) reported the first three confirmed cases ^4^. As of July 31, 2020, IEDCR confirmed 234,889 COVID-19 cases in Bangladesh, including 3083 related deaths with a Case Fatality Rate of 1.31% ^5^.

The COVID-19 pandemic has caused various challenges in Bangladesh's healthcare system. One of the biggest challenges is the spread of COVID-19 infections among frontline doctors ^6^. Up to July 29, 2020, about 2453 doctors have been infected ^7^, and 69 doctors have died ^8^ because of COVID-19 infection in Bangladesh. The mortality rate due to COVID-19 among doctors in Bangladesh is about 4%, which is the highest in the world among doctors ^9^, and this rate is also higher than that of Bangladesh's national mortality rate for COVID-19 ^5^.

In addition to the surge of COVID-19 infection, the pandemic has caused mental health problems to rise among doctors in Bangladesh. Mental health problems during pandemics are common, and evidence has shown that the severe acute respiratory syndrome (SARS), Middle East respiratory syndrome (MERS), and H1N1 pandemics also impacted the mental health condition of healthcare workers ^10– 12^. A study showed that frontline healthcare workers feel tremendous mental pressure during a pandemic because of the diminution of personal protection equipment, extensive media reportage, lack of treatment resources, increasing pattern of cases, death tolls, tremendous workload and social stigmatization ^13^. Recently, studies from Singapore, India, Greece and China have reported mental health issues of healthcare workers during the current rapidly evolving situation ^14– 17^. Besides all of these country-wise evidence, a case study of Bangladesh also reported an incident of suicide at a hospital due to fear of COVID-19. It was alleged that the suicide was committed because the victim was not treated by the health care professionals as they suspected the person was infected with COVID-19 ^18^. Again, another cross-sectional study reported COVID-19 suicidal behavior among the health professionals, and no comparable difference elucidated when compared with the general population ^19^. However, these two Bangladeshi studies reported suicidal behavior and/or fear of infection that prompted us to evaluate the other most commonly studied symptoms like anxiety, depression, and sleep disturbance, along with fear of COVID-19 in frontline doctors.

Bangladesh is a lower-middle-income country where doctors have to provide services in an overburdened, understaffed, and insufficiently equipped setting due to massive shortage and disproportionate distribution of skilled health workers, which causes unusual mental stress ^20^. Despite the challenges in their workplaces, during the COVID-19 pandemic Bangladeshi doctors have shown their competency and professionalism in providing the best care to the country's people. As part of their responsibility, they have to expose themselves to the risk of COVID-19 infection for the benefit of the mass population ^21^. It is speculated that the risk of infection and professional stress has gradually worsened the mental health condition of doctors in Bangladesh as they are facing stigmatization, fear of spreading the infection to family members and fear of being isolated. Currently, there is no evidence in support of this assumption. Therefore, we conducted this study to evaluate the psychological burden among Bangladeshi frontline doctors during the COVID-19 pandemic. To assess psychological symptoms, we quantified the magnitude of anxiety, depression, sleep disturbance and fear of COVID-19. Besides, we explored the associated factors influencing the psychological outcome. The findings of the study could be used to identify potential gaps in practice that would need interventions.

## Methods

### Study design and participants

We conducted an online cross-sectional study among doctors working at different clinical settings to treat patients, either suspected or confirmed COVID-19 cases, during the pandemic. Participants’ recruitment was completed by convenience sampling. Doctors from the professional and personal networks of the researchers were initially contacted through Facebook messenger and email. Doctors who showed interest were invited to participate in the study through an online questionnaire. We excluded doctors who did not complete intern training after graduation or were not involved in direct patient care.

A total of 370 frontline doctors took part in the study over two months from 1 ^st^ April to 30 ^th^ May 2020. Sample size was determined using the prevalence of anxiety, depression, insomnia, and distress among China's healthcare workers during the COVID-19 pandemic ^13^. The highest sample number was taken using the prevalence of depression (31.8%) in the aforementioned study, i.e. 370 respondents.

### Data collection

We circulated the questionnaire through online among the interested participants after the Government of Bangladesh confirmed community transmission in Bangladesh on March 28, 2020 ^22^. Data were collected by an online self-administered semi-structured questionnaire using the Google survey platform ^23^. The questionnaire link was sent to participants electronically through Facebook and email.

***Questionnaire content.*** The online questionnaire collected data on sociodemographic factors (age, gender, marital status, education, occupation), health service-related factors (the type of service, working place, professional designation, service level of health system, number of days of service provided, shifting duty or not, resource of working place), psychological parameters (anxiety, depression, sleep disturbance, fear), co-morbid conditions (diabetes, hypertension, asthma, chronic obstructive pulmonary disease, heart disease, chronic kidney disease, thyroid disorder), high-risk behavior as defined by tobacco use, and the living area of the physician where at least one COVID-19 case had been confirmed by the local authority.

The questionnaire was pre-tested before the final administration to detect any inconsistency and biases. To pre-test, 10 men and 10 women frontline doctors were selected randomly using the inclusion criteria (MBBS degree with completed intern training) and the questionnaire was sent to them through an online platform (Facebook messenger and email). The objective and importance of pre-testing were added with the questionnaire as an explanatory note. The researchers also informed that participation of the respondents was voluntary and they have the right to withdraw themselves at any time or refuse to answer any question. The collected responses were analyzed and interpreted based on the following: trends in responses; fundamental flaws with the design or format; attractiveness; comprehension; acceptance; and relevance.

***Instruments used to assess psychological symptoms.*** Anxious and depressive symptoms were assessed via the Patient Health Questionnaire-4 (PHQ-4) ^24^, which was an ultra-brief self-report questionnaire with a 2-item anxiety scale, named Generalized Anxiety Disorder 2-item (GAD-2), and a 2-item depression scale, named Patient Health Questionnaire 2-item (PHQ-2). Its reliability was acceptable and confirmed by a study as: PHQ-4 (Cronbach’s α=0.78), PHQ-2 (Cronbach’s α=0.75), and GAD-2 (Cronbach’s α=0.82) ^24^. The total score was determined by adding the scores of each of the four items as 0, 1, 2, and 3. Scores were rated as normal (0–2), mild (3–5), moderate (6–8), and severe (9–12). Total score ≥3 for the first two questions suggested anxiety. Total score ≥3 for the last two questions suggested depression ^24^.

Sleep disturbance was assessed via a two-item version of the Sleep Condition Indicator (SCI-02), an ultra-short clinical rating scale, which can be used to rapidly screen for insomnia in routine clinical practice ^25^. Each item was scored on a 5-point scale as 0, 1, 2, 3, 4. By adding the item scores, the SCI total score was obtained, ranging from 0 to 8. A higher score means better sleep. This tool showed an acceptable level of Cronbach’s α and the Spearman–Brown correlation at the point of 0.74. Again, the test-retest reliability (r) and intraclass correlation coefficient (ICC) in a sample repeating the test from 12 hours up to 7 days were r = 0.68 and ICC = 0.68, respectively ^25^. To quantify the magnitude of severity, we categorized the sleep disturbance using percentiles of the SCI-02 score as follows: good sleep condition (score ≥75th percentile, score ≥7), moderate sleep condition (score ≥25th percentile and <75th percentile, score 3–6) and insomnia (score <25th percentile, score 0–2). Here, the cut-off value of insomnia was kept the same as DSM-5 threshold criteria ^26^.

The Fear of Coronavirus-19 Scale (FCV-19S) was used to measure one’s fear of COVID-19 ^27^. The FCV-19S consists of 7 items. Participants were asked to rate their agreement with each statement on a 5-point scale from ‘1 - strongly disagree’ to ‘5 - strongly agree'. A higher score indicated greater fear. Recently, this instrument was validated among the Bangladeshi population ^28^. Currently, the FCV-19S has no classification of severity, and hence, we developed a severity scale using percentiles of FCV-19S score as follows: mild (score ≤25th percentile, score ≤17), moderate (score >25th percentile and <75th percentile, score 18 to 23) and severe (score ≥75th percentile, score ≥24).

### Statistical analysis

The data were entered in a pre-designed Microsoft Office Excel format, which was imported later into the software Statistical Package for Social Science version 20.0 for Windows (SPSS, Inc. Chicago. IL.USA). All the estimates of precision were presented at a 95% confidence interval (CI). Descriptive analysis included mean, standard deviation (SD), frequencies, and percentages. Background information (sociodemographic and professional) and the magnitude of psychological outcomes were presented using frequencies and percentages. The score of the instruments was presented using the mean with SD.

The associated factors of psychological outcomes were determined using multinomial logistic regression analysis. To find the factors that influenced the psychological outcomes, first, we run univariate analysis. Variables that showed p ≤0.25 in the univariate analysis were examined as an independent variable in the logistic regression ^29, 30^. We calculated odds ratios (OR) and 95% CI for each independent variable for multiple logistic regression analysis. In the regression table, factors that had OR >1 were presented for each outcome variable. We ensured no multicollinearity presence using the variance inflation factor (VIF) to run the regression analysis. The statistical tests were considered significant (2-sided) at a level of p ≤0.05.

### Ethical approval

The Ethical Review Committee of Bangladesh University of Health Sciences approved the study (identification number: BUHS/ERC/20/16).

An information and consent form ( *Extended* data ^23^) to take part in the study and for the publication of the participant’s anonymized information was provided prior to the questionnaire. Completion of the questionnaire implied consent.

## Results

**Figure 1.**
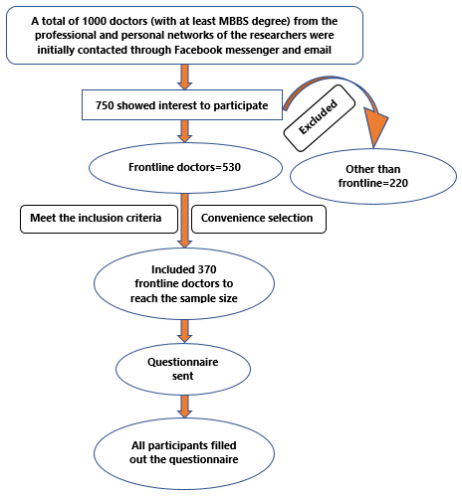
Flowchart for the enrollment and follow-up of participants.

As shown in Figure 1, 1000 individuals were contacted initially and 370 were included in the study after exclusion. 

### Demographic characteristics and health status of the doctors

The mean (SD) age of the doctors was 30.5 (4.4) years. Most of them were men (60.3%) and married (66.8%). A total of 69.5% had been living in areas that were affected by the COVID-19 outbreak. About a quarter of participants (24.8%) had been suffering from at least one chronic disease; the proportion of more-than-one chronic diseases was 4.3%. The most commonly reported chronic disease was chronic bronchial asthma (15.9%). Table 1 presents the detailed demographic and health-related characteristics of the study participants.

**Table 1.** Demographic and health-related characteristics of frontline doctors of Bangladesh (n= 370).

Characteristics	*n* (%)	95% CI
Age categories (years)		
< 30	185 (50)	44.9 – 55.1
≥ 30	185 (50)	44.9 – 55.1
Gender		
Men	223 (60.3)	55.3 – 65.3
Women	147 (39.7)	34.7 – 44.7
Marital status		
Married	247 (66.8)	62 – 71.6
Other	123 (33.2)	28.4 - 38
Residence in COVID-19 affected area	257 (69.5)	64.8 – 74.2
Current tobacco user	47 (12.7)	9.3 – 16.1
Presence of chronic disease	92 (24.8)	20.4 – 29.2
Number of chronic diseases		
At least one	76 (20.5)	16.4 – 24.6
More than one	16 (4.3)	2.2 – 6.4
Proportion of chronic diseases		
Chronic bronchial asthma	59 (15.9)	12.2 – 19.6
Hypertension	29 (7.8)	5.1 – 10.5
Diabetes	11 (3)	1.3 – 4.7
Others	13 (3.5)	1.6 – 5.4

COVID-19, coronavirus 2019

### Professional and work-related characteristics of the doctors

More than half of the total doctors (56.5%) had a Bachelor's (MBBS) degree, which is the entry-level degree for medical doctors in Bangladesh, and 19.7% had post-graduation degrees. The rest were post-graduate students (23.8%). The majority was employed in the private sector (55.4%), followed by the government sector (30.3%). Most of the doctors' primary working settings were a hospital (54.3%), and most of them worked at tertiary level healthcare settings (32.2%). The majority of the doctors had shifting duties (69.5%) and worked in a low resource setting (70.5%). On average, they worked five days a week during the pandemic ( Table 2).

**Table 2.** Professional background of frontline doctors of Bangladesh (n= 370).

Background	*n* (%)	95% CI
Educational qualification		
Bachelor (MBBS) degree	209 (56.5)	51.4 – 61.6
Post-graduate student	88 (23.8)	19.5 – 28.1
Post-graduate degree	73 (19.7)	15.6 – 23.8
Service types		
Private	205 (55.4)	50.3 – 60.5
Government	112 (30.3)	25.6 – 35
Other	53 (14.3)	10.7 – 17.9
Designation		
Medical officer/Assistant surgeon	222 (60)	55 – 65
Registrar to Professor	55 (14.9)	11.3 – 18.5
General practitioner	54 (14.6)	11 – 18.2
Other	39 (10.5)	7.4 – 13.6
Primary working place		
Private chamber/Diagnostic centre	36 (9.7)	6.7 – 12.7
Medical College	66 (17.8)	13.9 – 21.7
Hospital	201 (54.3)	49.2 – 59.4
Other	67 (18.1)	14.2 – 22
Service level		
Primary (Upazila & below)	95 (25.7)	21.3 – 30.1
Secondary (district hospital)	31 (8.4)	5.6 – 11.2
Tertiary (Medical college hospital)	119 (32.2)	27.5 - 37
Specialized	91 (24.6)	20.2 - 29
Other	34 (9.2)	6.3 – 12.1
Rotating/shifting duty	257 (69.5)	64.8 – 74.2
Service day/week *	5.2 (1.5)	
Resource of working health centre		
Sufficient	109 (29.5)	24.9 – 34.1
Insufficient	261 (70.5)	65.9 – 75.1

*Representing mean and standard deviation. MBBS, Bachelor of Medicine and Bachelor of Surgery; NGO, non-government organization; DG, directorate general; CI, confidence interval.

### Psychological burden of COVID-19 outbreak among the doctors

The detailed result of psychological status is presented in Table 3. The mean (SD) score of PHQ4, GAD-2 score and PHQ-2 score were 4.5 (2.9), 2.3 (1.8) and 2.2 (1.6), respectively. Considering the total score of PHQ4, about 73% of doctors had anxiety and/or depression, of which the majority were affected by mild anxiety and/or depression (39.2%). Separately, the first two (GAD-2) and successive two (PHQ-2) items of PHQ-4 identified that 36.5% of the doctors had anxiety, and 38.4% had depression. Here, the mean (SD) score of SCI-2 and FCV-19S were 5 (2.4) and 20.3 (6.1), respectively. Moreover, in the SCI-2 score, 18.6% of the doctors were found to be insomniac. Furthermore, the FCV-19S identified that 31.9% and 37.6% of the physicians had a severe and moderate level of fear regarding the COVID-19 pandemic, respectively.

**Table 3.** Psychological burden among frontline doctors of Bangladesh during the COVID-19 pandemic (n= 370).

Variables	*n* (%)	95% CI
**Anxiety and depression**		
Total PHQ-4 score *	4.5 (2.9)	
Normal (0 – 2)	100 (27)	22.5 – 31.5
Mild (3 – 5)	145 (39.2)	34.2 – 44.2
Moderate (6 – 8)	84 (22.7)	18.4 - 27
Severe (9 – 12)	41 (11.1)	7.9 -14.3
Total GAD-2 score (items 1, 2 of PHQ-4) *	2.3 (1.8)	
Total PHQ-2 score (items 3, 4 of PHQ-4) *	2.2 (1.6)	
Presence of anxiety	135 (36.5)	31.6 – 41.4
Presence of depression	142 (38.4)	33.5 – 43.3
**Sleep disturbance**		
Total SCI-02 score *	5 (2.4)	
Insomnia	69 (18.6)	14.6 – 22.6
Moderate sleep condition	170 (45.9)	40.8 – 51
Good sleep condition	131 (35.4)	30.5 – 40.3
**Fear of COVID-19**		
Total FCV-19S score *	20.3 (6.1)	
Mild	113 (30.5)	25.8 – 35.2
Moderate	139 (37.6)	32.7 – 42.5
Severe	118 (31.9)	27.2 – 36.6

*Representing mean and standard deviation. PHQ, Patient Health Questionnaire; GAD-2, Generalized Anxiety Disorder 2-item; SCI, Sleep Condition Indicator; COVID-19, coronavirus 2019; FCV-19S, fear of coronavirus 2019 scale; CI, confidence interval

### Predictors of the poor psychological status of the doctors

The univariate analysis (Chi-square test) showed association between PHQ4 (anxiety and/or depression) categories and several factors including gender (p=0.03), inadequate resources (p<0.001), presence of chronic disease (p=0.001), number of chronic diseases (p=0.003), asthma (p=0.002), and hypertension (0.005) ( Table 4). However, in the multinomial regression model, only inadequate resources in a working setting was found to be a significant predictor for severe (OR:2.99, 95% CI: 1.25- 7.15, p=0.01), moderate (OR:5.30, 95% CI: 2.54- 11.09, p<0.001), and mild (OR:2.28, 95% CI: 1.33-3.92, p=0.003) anxiety and/or depression controlling gender, presence of chronic disease, number of chronic diseases, asthma, and hypertension ( Table 5).

**Table 4.** Factors associated with anxiety and/or depression, sleep disturbance, and fear of COVID-19 among frontline doctors of Bangladesh during the COVID-19 pandemic, using the Chi-square test (n=370).

Factors	Categories	Anxiety and/or depression using PHQ4					Sleep disturbance using SCI-02				Fear of COVID-19 using FCV-19S			
		Severe	Moderate	Mild	Normal	*p*-value	Insomnia	Moderate	Good	*p*-value	Severe	Moderate	Mild	*p*-value
Gender	Men	19	46	87	71	**0.03**	38	105	80	061	55	83	85	**<0.001**
	Women	22	38	58	29		31	65	51		63	56	28	
Age	≥30 years	20	35	72	58	0.18	21	91	73	**0.001**	51	70	64	0.12
	<30 years	21	49	73	42		48	79	58		67	69	49	
Working area	Hospital	17	42	80	62	0.13	28	95	78	**0.01**	54	71	76	**0.002**
	Medical college	12	18	26	10		22	28	16		30	20	16	
	Other	12	24	39	28		19	47	37		34	48	21	
Shifting duty	Yes	32	65	98	62	0.07	51	126	80	**0.04**	86	97	74	0.47
	No	9	19	47	38		18	44	51		32	42	39	
Adequate resource	Yes	9	12	41	47	**<0.001**	18	42	49	**0.05**	28	37	44	**0.03**
	No	32	72	104	53		51	128	82		90	102	69	
Residence in COVID affected area	Yes	30	62	103	62	0.28	50	130	77	**0.004**	84	94	79	0.82
	No	11	22	42	38		19	40	54		34	45	34	
Chronic disease	Yes	19	25	31	17	**0.001**	20	44	28	0.45	33	35	24	0.49
	No	22	59	114	83		49	126	103		85	104	89	
Number of chronic diseases	≥2	6	4	2	4	**0.003**	4	2	10	**0.01**	6	7	3	0.58
	≤1	35	80	143	96		65	168	121		112	132	110	
Asthma	Yes	13	19	18	9	**0.002**	15	31	13	**0.05**	24	24	11	0.08
	No	28	65	127	91		54	139	118		94	115	102	
Hypertension	Yes	9	5	9	6	**0.005**	6	8	15	0.09	9	13	7	0.65
	No	32	79	136	94		63	162	116		109	126	106	

PHQ, Patient Health Questionnaire; SCI, Sleep Condition Indicator; COVID-19, coronavirus 2019; FCV-19S, fear of coronavirus 2019 scale. *p*-value significant at the threshold of ≤0.05

**Table 5.** Factors determining the psychological burden of the COVID-19 pandemic among frontline doctors of Bangladesh, using multinomial logistic regression analysis (n= 370).

Associated factors of anxiety and/ or depression	Severity of anxiety and/or depression using PHQ4 instrument (Ref. normal)								
	*Mild*			*Moderate*			*Severe*		
	*p*-value	OR	95% CI	*p*-value	OR	95% CI	*p*-value	OR	95% CI
Inadequate resource (Ref. adequate resource)	**0.003**	**2.28**	**1.33-3.92**	**<0.001**	**5.30**	**2.54- 11.09**	**0.014**	**2.99**	**1.25- 7.15**
Suffering from NCD (Ref. no NCD)	0.91	0.93	0.25-3.45	0.75	1.26	0.31-5.14	0.80	1.24	0.23-6.67
Asthma present (Ref. no Asthma)	0.41	1.86	0.43-8.02	0.22	2.61	0.57- 11.92	0.13	3.83	0.69- 21.40
HTN present (Ref. no HTN)	0.26	3.25	0.42-25.48	0.81	0.71	0.04- 11.78	0.13	6.71	0.57- 79.27
**Associated factors of sleep** **disturbance**	**Severity of sleep disturbance using SCI-02 instrument (Ref. good sleep condition)**								
	*Moderate sleep condition*					*Insomnia*			
	*p*-value	OR		95% CI		*p*-value	OR		95% CI
Men (Ref. women)	0.39	1.25		0.75-2.07		0.009	0.41		0.21-0.80
Shifting duty (Ref. no shifting duty)	0.007	2.21		1.24-3.94		0.10	1.87		0.89-3.93
Inadequate resource (Ref. adequate resource)	0.02	1.85		1.08-3.16		0.50	1.27		0.63-2.53
Living in COVID-19 affected area (Ref. not living in COVID-19 affected area)	0.001	2.38		1.41-4.01		0.17	1.59		0.82-3.10
Asthmatic (Ref. no asthma	**0.004**	**3.33**		**1.47-7.54**		**0.004**	**4.06**		**1.57-10.51**
Working in a medical college (Ref. working in other institution)	0.78	0.89		0.39-2.03		0.12	2.07		0.82-5.23
**Associated factors of fear**	**Severity of fear using FCV-19S (Ref. mild fear)**								
	*Moderate fear*					*Severe fear*			
	*p*-value	OR		95% CI		*p*-value	OR		95% CI
Working in a medical college (Ref. working in other institution)	0.16	0.54		0.23-1.27		0.55	1.30		0.55-3.07
Inadequate resource (Ref. adequate resource)	**0.034**	**1.82**		**1.05-3.16**		**0.035**	**1.90**		**1.05-3.47**

PHQ, Patient Health Questionnaire; SCI, Sleep Condition Indicator; COVID-19, coronavirus 2019; FCV-19S, fear of coronavirus 2019 scale; Ref., reference. p-value significant at the threshold of ≤0.05

Regarding sleep disturbance, the univariate analysis found age (p=0.001), working area (p=0.01), shifting duty (p=0.04), inadequate resources (p=0.05), residence in a COVID-19 affected area (p=0.004), number of chronic diseases (p=0.01), and asthma (p=0.05) as the associated factors ( Table 4). Among the associated factors, only asthma was found as a significant predictor of insomnia (OR: 4.06, 95% CI: 1.57-10.51, p=0.004) and moderate sleep condition (OR: 3.33, 95% CI: 1.47-7.54, p=0.004) controlling all other associated factors in a regression model. In addition, shifting duty (OR: 2.21, 95% CI: 1.24-3.94, p=0.007), inadequate resources (OR: 1.85, 95% CI: 1.08-3.16, p=0.02), and living in a COVID-19 affected area (OR: 2.38, 95% CI: 1.41-4.01, p=0.001) were also found as significant predictors for moderate sleep condition ( Table 5). 

Regarding fear of COVID-19, the univariate analysis found gender (p<0.001), primary working area (p=0.002), and inadequate resources (p=0.03) as associated factors ( Table 4). However, in multinomial regression analysis, only inadequate resources was found as the significant predictor for severe (OR: 1.90, 95% CI: 1.05-3.47, p=0.03) and moderate (OR: 1.82, 95% CI: 1.05-3.16, p=0.03) fear of COVID-19 ( Table 5). 

## Discussion

The study aimed to assess the psychological burden of frontline doctors in Bangladesh during the COVID-19 pandemic, and factors that predict their psychological status. The study identified that anxiety, depression, insomnia, and fear related to the COVID-19 outbreak are common among frontline doctors of Bangladesh during this unprecedented time. The paucity of resources for providing care to patients in workplaces was found as the single most common predictor for poor psychological status. In addition, having shifting duty, living in a COVID-19 affected area, and the presence of asthma predicted poor quality of sleep among the frontline doctors.

A considerable proportion of frontline doctors in Bangladesh has experienced psychological symptoms due to the COVID-19 pandemic. The burden of psychological symptoms is higher than the burden of symptoms among healthcare workers of China, Singapore and India during the COVID-19 pandemic ^14, 15, 17^. A meta-analysis study from China has presented the pooled prevalence of depression (22.8%), anxiety (23.2%), and insomnia (38.9%) ^17^. Compared to the pooled prevalence of symptoms in China, the current study has shown a higher proportion of depression and anxiety, but a lower proportion of insomnia among Bangladeshi doctors. Furthermore, the prevalence of anxiety and depression were reported as 14.4% and 9%, respectively, in Singapore ^15^ and 17.1% and 12.4%, respectively, in India ^14^, which are also lower than the magnitude of anxiety and depression observed among Bangladeshi doctors in this study. The burden of psychological symptoms in the current study is also higher than the burden of psychological symptoms among China's general population during the pandemic ^31^. Similarly, the burden of depression in our study is also higher than the depression reported by another study among the general population of Bangladesh ^32^. Moreover, a comparison with mental health symptoms (anxiety 77.4%, depression 74.2%, and sleep problems 52.3%) among health workers during SARS pandemics in Taiwan shows a lower burden of psychological symptoms in Bangladesh during COVID-19 pandemics ^10^. It is noteworthy that there are variations in the methods of measuring psychological symptoms across the studies.

Many underlying factors for mental health problems among frontline health workers during the pandemic situation have been reported in the literature, including gender, age, living in a rural area, poor social support, poor self-efficacy, profession, place of work, disruption of routine clinical practice, fear of potential destabilization of health services, the sense of loss of control, having organic disease, and being at risk of contact with a patient with COVID-19 ^13, 16, 31, 33– 35^. Among all the reported causes, COVID-19 can be an independent risk factor for healthcare workers' poor mental health ^33^. In Bangladesh, the burden of COVID-19 is among the top 20 countries in the world. Along with the general population, frontline health workers have also been overwhelmed by the surge of infection. It has been reported that doctors in Bangladesh have been experiencing the highest infection and mortality in the world due to the virus ^9^. Experts have suggested that lack of infection control measures, monitoring, proper management at hospitals, inappropriate use and disposal of safety gear, and lack of training for dealing with patients with COVID-19 are contributing to the highest infection and mortality of the doctors ^9^. It is also believed that COVID-19 infection and its underlying causes contribute to the doctors' poor mental health condition.

The current study identified several factors that contribute to the burden of psychological symptoms among Bangladeshi doctors - the paucity of resources in the workplace is the most significant. Limited resources in the workplace include materials, trained workforce or any other things that are required to provide services. The current study has confirmed the association of inadequate resources with the poor psychological status of doctors. Inadequate resources such as masks, sanitizer, and personal protective equipment (PPE) in workplaces increase the chance of getting COVID-19 infection and can cause profound psychological pressure on frontline doctors. The lack of resources in workplaces in Bangladesh has been widely reported in news media ^36^. The news media has reported inadequate and inappropriate PPE as a cause of widespread COVID-19 infection among healthcare professionals in Bangladesh ^36^. However, Bangladesh is not the only country that faced a shortage of resources during the pandemic. The shortage of such resources has also been reported in many other countries because of the distorted supply chain across the world ^37^. Lack of resources is also considered as a cause of poor psychological status among healthcare workers in many countries during the pandemic ^16, 38^. Experts have recognized sufficient resources as an essential factor for healthcare professionals to be resilient during an unprecedented time ^39^. 

The lack of skilled and trained workforce in hospitals is another underlying cause of the high burden of psychological symptoms among frontline doctors in Bangladesh. Amid the workforce shortage, frontline doctors have to do long shifting duties for a certain period and then stay in quarantine for 14 days before they return to work. This atypical work schedule for doctors has been introduced to reduce the frequency of exposure to COVID-19 virus in workplaces. However, it is believed that the long shifting duties and being isolated during quarantine may have triggered mental health problems among doctors. The current study has found that those who did shifting duties were more likely to have sleep problems linked with poor mental health. Although identifying links between the quarantine period and poor mental health was not a scope of the current study, other studies have confirmed the link between quarantine period and mental health during this pandemic ^40^.

The current study is the first study in Bangladesh that provides the burden and associated factors for doctors' poor mental health outcome during the COVID-19 pandemic. There are some limitations in the study. As it is a cross-sectional study, causal relation could not be established. Thus, the study presents the factors linked with the psychological outcomes as associated factors. Moreover, the study is an online-based questionnaire. Therefore, the possibility of selection bias cannot be ruled out. Again, a small sample size limited the generalization of the study findings. The participants of the study were mainly young doctors. This happened because the younger population is more exposed to online platforms than the elderly. However, a recent review has shown that younger doctors are more affected by psychological symptoms than elder doctors ^34^. Thus, the study has reflected evidence of the high-risk group of doctors for a psychological problem.

## Conclusions

A high burden of COVID-19 related anxiety, depression, sleep disturbance, and fear among Bangladeshi frontline doctors demands policymakers' immediate attention to take appropriate preventive measures. An appropriate risk-reduction strategy should be developed and implemented to reduce the risk of getting COVID-19 infection. In addition, the supply of adequate PPE and the development of a trained workforce with infection control skills need to be considered to reduce the psychological impact. The substantial burden of different mental health outcomes elucidated in the current study demands mental health counsellors in hospital settings where appropriate. Considering low resource settings, this strategy could be implemented at least in COVID-19 dedicated hospitals in Bangladesh.

## Data availability

### Underlying data

Zenodo: The psychological burden of the COVID-19 pandemic and its associated factors among the frontline doctors of Bangladesh: A cross-sectional study, http://doi.org/10.5281/zenodo.4110337 ^41^.

Data are available under the terms of the Creative Commons Zero "No rights reserved" data waiver (CC0 1.0 Public domain dedication). 

### Extended data

Zenodo: The Psychological Burden of the COVID-19 Pandemic and Its Associated Factors among the Frontline Doctors of Bangladesh: A Cross-sectional Study-Extended Data, https://doi.org/10.5281/zenodo.4058715 ^23^.

This project contains the following extended data within the file ‘Extended data file.pdf’:

— Consent form (English Version)
— Questionnaire (English Version)

### Reporting guidelines

Zenodo: STROBE checklist for ‘The Psychological Burden of the COVID-19 Pandemic and Its Associated Factors among the Frontline Doctors of Bangladesh: A Cross-sectional Study’, https://doi.org/10.5281/zenodo.4062170 ^42^.

Data are available under the terms of the Creative Commons Zero "No rights reserved" data waiver (CC0 1.0 Public domain dedication).
