# Supplementary material for: Psychological burden of the COVID-19 pandemic and its associated factors among frontline doctors of Bangladesh: a cross-sectional study
Source: F1000Res. 2021 Jan 11;9:1304. Originally published 2020 Nov 6. [Version 3] doi: 10.12688/f1000research.27189.3 (PMC7783536; doi:10.12688/f1000research.27189.3)
Supplement: Peer review history

# Peer review: Reviewer response for version 2

**DOI:** 10.5256/f1000research.31333.r76228

**Published:** 2021-01-04

**Authors:** Mohammed Mamun

**Affiliations:** 1 Centre for Health Innovation, Networking, Training, Action and Research - Bangladesh (CHINTA Research Bangladesh), Dhaka, Bangladesh

Authors did a great job incorporating and responding to the comments. By the way, I have only a request to correct before final approval: 

 "However, these two Bangladeshi studies reported a single parameter of mental health (suicidal behavior) that prompted us to evaluate the other most commonly studied symptoms like anxiety, depression, sleep disturbance, and fear of COVID-19 in frontline doctors." - Please note, one of the studies is a case report, where the patient committed suicide, not any HCWs. The case reports definitely emphases the fear of infection existing in the healthcare setting. I think this line need to be rewritten.

Is the work clearly and accurately presented and does it cite the current literature?

Yes

If applicable, is the statistical analysis and its interpretation appropriate?

Partly

Are all the source data underlying the results available to ensure full reproducibility?

Yes

Is the study design appropriate and is the work technically sound?

Yes

Are the conclusions drawn adequately supported by the results?

Yes

Are sufficient details of methods and analysis provided to allow replication by others?

Yes

Reviewer Expertise:

Psychiatric Epidemiology

I confirm that I have read this submission and believe that I have an appropriate level of expertise to confirm that it is of an acceptable scientific standard.

# Response

**Published:** 2021-01-04

**Authors:** Lingkan Barua

**Affiliations:** Bangladesh University of Health Sciences (BUHS), Bangladesh

Thank you for the valuable comment. We revised the sentence according to your suggestion as "However, these two Bangladeshi studies reported suicidal behavior and/or fear of infection that prompted us to evaluate the other most commonly studied symptoms like anxiety, depression, and sleep disturbance along with fear of COVID-19 in frontline doctors”.

# Peer review: Reviewer response for version 1

**DOI:** 10.5256/f1000research.30039.r74583

**Published:** 2020-12-07

**Authors:** Ganiyu Adeniyi Amusa

**Affiliations:** 1 Department of Medicine, Jos University Teaching Hospital, Jos, Nigeria

**General Comments: **

- An interesting study, well written, appropriate methodology/statistical analysis and evidence-based conclusion derived from the results. The tables are appropriate, clear and precise, no redundancy observed. The references are appropriate, recent and properly cited; no redundancy noted in the referencing

**Abstract:**

- Well written, established the basis for the study and highlighted main findings of the study. The study revealed that 36.5% had anxiety, 38.4% had depression, 18.6% had insomnia, and 31.9% had fear of COVID-19. Inadequate resources in the workplace were found as the single most significant predictor for all psychological outcomes.

**Introduction:**

- The origin of the pandemic was well captured as well as the attendant societal and individual consequences. The story of the pandemic in Bangladesh was also well captured and the basis for the need to do the study was adequately elucidated bearing in mind the unique characteristics of the Bangladesh community.

**Methods:**

- The methodology was described in detail, clear enough and can be reproduced elsewhere. The questionnaire content was detailed and appropriate and was well able to meet the objectives of the study. The instruments used were appropriate - Patient Health Questionnaire-4 (PHQ-4) to assess anxious and depressive symptoms, Sleep Condition Indicator (SCI-02) to assess sleep disturbance and Fear of Coronavirus-19 Scale (FCV-19S) was used to measure one’s fear of COVID-19. The cut-off used and methods of determining the score are appropriate and scientific. The statistics (descriptive and inferential) used were appropriate for the results and conclusion reached.

**Results:**

- The results were presented in appropriate formats with clear details. No redundancy was observed.

**Discussion:**

- This was extensive and relevant examples were used in the discussion as related to the findings of the study. The result of a metanalysis of similar studies was comparable to that of the study. Similar findings were obtained in India, Singapore and Taiwan.

**Conclusion:**

- The study concluded that a high burden of COVID-19 related anxiety, depression, sleep disturbance, and fear existed among Bangladeshi frontline doctors. This demands policymakers' immediate attention to take appropriate preventive measures. The substantial burden of different mental health outcomes elucidated in the current study demands mental health counselors in hospital settings where appropriate.

Is the work clearly and accurately presented and does it cite the current literature?

Yes

If applicable, is the statistical analysis and its interpretation appropriate?

Yes

Are all the source data underlying the results available to ensure full reproducibility?

Yes

Is the study design appropriate and is the work technically sound?

Yes

Are the conclusions drawn adequately supported by the results?

Yes

Are sufficient details of methods and analysis provided to allow replication by others?

Yes

Reviewer Expertise:

NA

I confirm that I have read this submission and believe that I have an appropriate level of expertise to confirm that it is of an acceptable scientific standard.

# Response

**Published:** 2020-12-08

**Authors:** Lingkan Barua

**Affiliations:** Bangladesh University of Health Sciences (BUHS), Bangladesh

Dear reviewer, thank you for your positive observations regarding our manuscript.

# Peer review: Reviewer response for version 1

**DOI:** 10.5256/f1000research.30039.r74585

**Published:** 2020-11-24

**Authors:** Mohammed Mamun

**Affiliations:** 1 Centre for Health Innovation, Networking, Training, Action and Research - Bangladesh (CHINTA Research Bangladesh), Dhaka, Bangladesh

I would like to thank the authors for assessing such a cohort (i.e., Frontline fighters), as there is almost no study in Bangladesh to the cohort. Their studied mental health outcomes such as depression, anxiety, insomnia and fear of COIVD is appreciable. However, a few observations are provided herein, which may be considered.

- Instead of frontline doctors, frontline fighters may be used – because this word is widely used in the press media, and your article may get more visibility by using it. My comment is not mandatory as you only collected data from doctors, whereas fighters includes nurse, paramedics etc.
- after the start of community transmission of COVID-19 in the country – may I suggest to add the exact time frame?
- Recently, articles from Singapore, India, Greece and China – please replace the word ‘articles’ with ‘studies’.
- The authors precisely reported the relevance of the study (i.e., evaluating mental health suffering in doctors). However, I would like to suggest referring a case study reporting excessive fear of COVID-19 existing in the healthcare facilities in Bangladesh. Consequently, a non- COVID-19 patient committed suicide. I think this information can add an extra value to your study rationale. Ref: https://doi.org/10.1016/j.ajp.2020.102295
- Besides, there is a Bangladeshi case-control large scale study accessing suicidality can be discussed and compare what is new in your study. For example, the study got not statistical significance difference in suicidality across general people and healthcare professionals. Ref: https://doi.org/10.1016/j.heliyon.2020.e05259
- More information regarding sampling is needed. Please add scale reliability (e.g., Cronbach's alpha) as the scales are not validated in Bangla.
- The authors already mentioned, there is no cutoff score for Fear of COVID scale. However, they categorized it. It would be better if they provide how the category was considered – median?
- It is requested to report the mean and SD of fear of COVID (along with percentage; as it was done for depression and anxiety) as the scale has no cutoff score yet.
- Is not it better to report psychological burdens as “probable depression” instead of “depression”, because the scales are short version (although their cutoff scores had higher specificity and sensitivity than the original one; for example, PHQ-2 and PHQ-9)? Similar suggestion for fear of COVID. Besides, the authors may like to perform ANOVA and linear regression – not mandatory if they refer how the cutoff scores were categorized.
- Discussion is focused on the results. Discussing prevalence rate across countries is perfect, even the authors compared with the review article prevalence rate, which is appreciated. By the way, may I suggest to compare the Bangladeshi general people mental health problems rates, this may help the reader to be informed about how this study findings differ from the general people. Please refer Bangladeshi studies assessing the pandemic related psychological burdens (ref: https://doi.org/10.31234/osf.io/q4k5b).
- The current study is the first study in Bangladesh that provides the burden and associated factors for doctors’ poor mental health outcome during the COVID-19 pandemic. – I request to avoid the first word, because there is a published paper on suicidal behavior of the cohort.

Overall observations, the authors did a great job addressing mental health problems of the vulnerable cohort to virus infection, which may have influence in policy level. And the paper was written in a good flow, and also provided some of recommendations. Best of luck.

Is the work clearly and accurately presented and does it cite the current literature?

Yes

If applicable, is the statistical analysis and its interpretation appropriate?

Partly

Are all the source data underlying the results available to ensure full reproducibility?

Yes

Is the study design appropriate and is the work technically sound?

Yes

Are the conclusions drawn adequately supported by the results?

Yes

Are sufficient details of methods and analysis provided to allow replication by others?

Yes

Reviewer Expertise:

Psychiatric Epidemiology

I confirm that I have read this submission and believe that I have an appropriate level of expertise to confirm that it is of an acceptable scientific standard, however I have significant reservations, as outlined above.

# Response

**Published:** 2020-12-08

**Authors:** Lingkan Barua

**Affiliations:** Bangladesh University of Health Sciences (BUHS), Bangladesh

**Response to the Reviewer1**

 We would like to thank you for your overall comment on our submitted manuscript. Here we have added our responses. The changes have presented using track changes.

**(A) Initial Comments**

 I would like to thank the authors for assessing such a cohort (i.e., Frontline fighters), as there is almost no study in Bangladesh to the cohort. Their studied mental health outcomes such as depression, anxiety, insomnia and fear of COIVD is appreciable. However, a few observations are provided herein, which may be considered.

**Comment 1:**

 Instead of frontline doctors, frontline fighters may be used – because this word is widely used in the press media, and your article may get more visibility by using it. My comment is not mandatory as you only collected data from doctors, whereas fighters includes nurse, paramedics etc.

**Response 1:**

 Thanks for your valuable comment about our target population. To present the target population more precisely and specifically, the term ‘Frontline doctors’ is best suited and also aligned with the 2 ^nd^ sentence of the reviewer comment (“whereas fighters include nurse, paramedics etc”).

**Comment 2:**

 After the start of community transmission of COVID-19 in the country – may I suggest to add the exact time frame?

**Response 2:**

 Thanks for your valuable comment. The time frame has already mentioned in the two separate sections of the ‘Methods’ part. In the ‘Study design and participants’ section, paragraph 02, we mentioned-“A total of 370 frontline doctors took part in the study over two months from 1st April to 30th May 2020”. Again, in the ‘Data collection’ section, 1 ^st^ paragraph, and in the 1 ^st^ line we mentioned: “the Government of Bangladesh confirmed community transmission in Bangladesh on March 28, 2020”. So community transmission was confirmed on March 28, 2020, and after that, we started our data collection on 1 ^st^ April that ended on 30 ^th^ May 2020.

**Comment 3:**

 Recently, articles from Singapore, India, Greece and China – please replace the word ‘articles’ with ‘studies’.

**Response 3:**

 Corrected as suggested.

**Comment 4**

 The authors precisely reported the relevance of the study (i.e., evaluating mental health suffering in doctors). However, I would like to suggest referring a case study reporting excessive fear of COVID-19 existing in the healthcare facilities in Bangladesh. Consequently, a non- COVID-19 patient committed suicide. I think this information can add an extra value to your study rationale. Ref: https://doi.org/10.1016/j.ajp.2020.102295

**Response 4**

 We mentioned the case study as you suggested.

**Comment 5**

 Besides, there is a Bangladeshi case-control large scale study accessing suicidality can be discussed and compare what is new in your study. For example, the study got not statistical significance difference in suicidality across general people and healthcare professionals. Ref: https://doi.org/10.1016/j.heliyon.2020.e05259

**Response 5**

 We mentioned the study you suggested to discuss.

**Comment 6**

 More information regarding sampling is needed. Please add scale reliability (e.g., Cronbach's alpha) as the scales are not validated in Bangla.

**Response 6**

 We applied a convenience sampling technique that was clearly mentioned. Moreover, a flow chart was used to show the pool of samples from where the subjects were recruited based on inclusion criteria.

 Among the tools applied, only FCV-19S validated among the Bangladeshi population and we mentioned it in the manuscript. For PHQ-4 and SCI-02, as per your suggestion, we added the Cronbach's alpha as appropriate.

**Comment 7**

 The authors already mentioned, there is no cutoff score for Fear of COVID scale. However, they categorized it. It would be better if they provide how the category was considered – median?

**Response 7**

 We have already mentioned that we used ‘PERCENTILES’ to categorize the severity of the scales that have not yet developed their severity grades (see in the section ‘Instruments used to assess psychological symptom’). For fear of COVID-19, the FCV-19S score was categorized using percentiles of “mild” (score ≤25th percentile, ≤17), “moderate” (score >25th percentile and <75th percentile, 18–23), and “severe” (score ≥75th percentile, ≥24).

 To convince the readers, in the revised version, we have elaborated the method of categorization adding the percentiles with each cut-off value. This method of categorization also applied for another health-related tool also (https://doi.org/10.1111/jdi.13331).

**Comment 8**

 It is requested to report the mean and SD of fear of COVID (along with percentage; as it was done for depression and anxiety) as the scale has no cutoff score yet.

**Response 8**

 We have added.

**Comment 9**

 Is not it better to report psychological burdens as “probable depression” instead of “depression”, because the scales are short version (although their cutoff scores had higher specificity and sensitivity than the original one; for example, PHQ-2 and PHQ-9)? Similar suggestion for fear of COVID. Besides, the authors may like to perform ANOVA and linear regression – not mandatory if they refer how the cutoff scores were categorized.

**Response 9**

 As the generic version of PHQ-2 avoided the term “probable” to represent depression and showed an acceptable level of reliability, we used ‘depression’ to align with the main paper (A 4-item measure of depression and anxiety: Validation and standardization of the Patient Health Questionnaire-4 (PHQ-4) in the general population, https://doi.org/10.1016/j.jad.2009.06.019).

 Regarding fear of COVID-19, we presented the severity using the scientific method as described above. As this severity scale has not yet developed and validated, hence we pretested it (as part of the whole questionnaire) in a small sample of frontline doctors as mentioned in the section entitled ‘Questionnaire content’.

 We used multinomial logistic regression based on the categorization of the tools as explained in the revised version and also stated above to respond to the reviewer inquiry.

**Comment 10**

 Discussion is focused on the results. Discussing prevalence rate across countries is perfect, even the authors compared with the review article prevalence rate, which is appreciated. By the way, may I suggest to compare the Bangladeshi general people mental health problems rates, this may help the reader to be informed about how this study findings differ from the general people. Please refer Bangladeshi studies assessing the pandemic related psychological burdens (ref: https://doi.org/10.31234/osf.io/q4k5b).

**Response 10**

 Thank you for your valuable input. As the suggested study is a pre-print version, we avoided it and cited a nation-wide study that reported psychological symptoms among the general population of Bangladesh (https://doi.org/10.1016/j.jad.2020.10.036). We compared the prevalence of depression as appropriate.

**Comment 11**

 The current study is the first study in Bangladesh that provides the burden and associated factors for doctors’ poor mental health outcome during the COVID-19 pandemic. – I request to avoid the first word, because there is a published paper on suicidal behavior of the cohort.

**Response 11**

 As per our knowledge, this study is absolutely first as it reported the most commonly reported psychological symptoms that were not previously reported by any study of Bangladesh among frontline doctors. We respect the comment of the reviewer and want to mention that the previous study assessed suicidal behavior, not the ‘anxiety, depression, fear, and sleep disturbance’. Moreover, this study solely conducted among frontline doctors (not intern doctors) who were involved in treating the confirmed or suspected cases of COVID-19.

**(D) Overall Comments**

 Overall observations, the authors did a great job addressing mental health problems of the vulnerable cohort to virus infection, which may have influence in policy level. And the paper was written in a good flow, and also provided some of recommendations. Best of luck.

**Response**

 Thanks for the overall comments.
